# Nurses’ Perception of Tension, Stress, and Pressure before and during the COVID-19 Pandemic: A Multicenter Serbian Study

**DOI:** 10.3390/healthcare12060663

**Published:** 2024-03-15

**Authors:** Milena Santric-Milicevic, Kristina Pavlekic, Zoran Bukumiric, Aleksandar Stevanovic, Dejan Nikolic, Bojana Matejic, Dragana Matanovic, Dusan Backovic, Goran Tulic, Relja Lukic, Dubravka Zivanovic, Sofija Radosavljevic, Vladimir Milovanovic, Marija Zdujic, Sanja Stankovic, Milika Asanin, Marija Zdravkovic, Ratko Tomasevic

**Affiliations:** 1 Institute of Social Medicine, Faculty of Medicine, University of Belgrade, 11000 Belgrade, Serbiabojana.matejic@med.bg.ac.rs (B.M.);; 2 Laboratory for Strengthening Capacity and Performance of Health System and Workforce for Health Equity, Faculty of Medicine, University of Belgrade, 11000 Belgrade, Serbia; 3 City Institute of Public Health in Belgrade, 11000 Belgrade, Serbia; kristina.pavlekic@zdravlje.org.rs; 4 Institute for Medical Statistics and Informatics, Faculty of Medicine, University of Belgrade, 11000 Belgrade, Serbia; zoran.bukumiric@med.bg.ac.rs; 5 Department of Physical Medicine and Rehabilitation, University Children’s Hospital, 11000 Belgrade, Serbia; denikol27@gmail.com; 6 Faculty of Medicine, University of Belgrade, 11000 Belgrade, Serbia; dragana.matanovic@med.bg.ac.rs (D.M.); tulic05@gmail.com (G.T.); reljalu@gmail.com (R.L.); dubravkazivanovic@yahoo.com (D.Z.); vmilovanovic1972@yahoo.com (V.M.); milika.asanin@med.bg.ac.rs (M.A.); sekcija.kardioloska@gmail.com (M.Z.); ratko.tomasevic@med.bg.ac.rs (R.T.); 7 Center for Physical Medicine and Rehabilitation, University Clinical Center of Serbia, 11000 Belgrade, Serbia; 8 Institute for Hygiene and Medical Ecology, Faculty of Medicine, University of Belgrade, 11000 Belgrade, Serbia; 9 Institute for Orthopaedic Surgery and Traumatology, University Clinical Center of Serbia, 11000 Belgrade, Serbia; 10 Clinic for Gynecology and Obstetrics, University Clinical Center of Serbia, 11000 Belgrade, Serbia; 11 Clinic of Dermatology and Venerology, University Clinical Center of Serbia, 11000 Belgrade, Serbia; 12 Department of Radiology, University Hospital Center “Dr Dragisa Misovic”, 11000 Belgrade, Serbia; radosavljevicsofija0304@gmail.com; 13 Department of Cardiac Surgery, University Children’s Hospital, 11000 Belgrade, Serbia; 14 Center for Medical Biochemistry, University Clinical Center of Serbia, 11000 Belgrade, Serbia; sanjast2013@gmail.com; 15 Faculty of Medical Sciences, University of Kragujevac, 34000 Kragujevac, Serbia; 16 Department of Cardiology, University Clinical Center of Serbia, 11000 Belgrade, Serbia; 17 Internal Medicine Clinic, University Clinical Hospital Center Bezanijska Kosa, 11000 Belgrade, Serbia; 18 Internal Medicine Clinic, University Clinical Hospital Center Zemun, 11000 Belgrade, Serbia

**Keywords:** COVID-19, healthcare management, mental health, nurses, intensive care units

## Abstract

The mental health of healthcare workers, especially the nursing staff in intensive care units, is crucial for the optimal functioning of healthcare systems during medical emergencies. This study implements a cross-sectional design to investigate the associations between nurses’ personal characteristics, workplace challenges, and job satisfaction with the increased perception of tension, stress, and pressure at the workplace (TSPW) before and during the COVID-19 pandemic. In 2021, we surveyed 4210 nurses from 19 intensive healthcare facilities in the capital of Serbia, Belgrade, and, at that time, collected data about their perceived TSPW before and during the COVID-19 pandemic. Our study identified six predictors of the increase in TSPW, as perceived by nurses: their work in COVID-19 infectious zones (OR = 1.446), exhaustion due to work under protective equipment (OR = 1.413), uncertainty and fear of infection (OR = 1.481), a high degree of superiors’ appreciation and respect (OR = 1.147), a high degree of patients’ attitudes (OR = 1.111), and a low degree of work autonomy (OR = 0.889). The study’s findings suggest that a solution to this issue is necessary to ensure that nurses are safe and able to alleviate the physical and mental strain that comes with prolonged use of protective equipment. Nurses on the frontline of the pandemic require better health protection, better conditions, and respect for their role. Strategies to promote mental health would help reduce nurses’ stress and increase job satisfaction.

## 1. Introduction

As of 20 May 2022, more than 6.27 million deaths worldwide have been registered involving Coronavirus Disease 2019 (COVID-19), with global excess mortality estimated at 14.9 million deaths [1]. Furthermore, the World Health Organization estimates that in the first eighteen months of the pandemic, between 80,000 and 180,000 health workers have died due to COVID-19 [2]. However, the high risk of contracting COVID-19 [3] was not the only challenge faced by health professionals during the current pandemic. A recent study points out that the mental health of health workers is at increased risk compared to the rest of the population [4,5,6], and this can further decrease the quality of health and nursing care [7]. In line with Watson’s theory of human caring, nursing practice involves compassion to ease patients’ and families’ suffering, promote healing and dignity, and expand the nurses’ self-actualization [8]. However, the stress associated with the job can lead to a loss of compassion for patients as well as an increase in errors in practice, negatively affecting the quality of care [9]. Moreover, job stress causes fatigue, ineffective activity, and lack of concentration, leading to negative behaviors and attitudes toward oneself, work, family, and patients [10]. The poor mental health of nursing professionals can negatively affect patient safety, and errors can be made, leading to the inability to provide the best quality of care performance [11]. Finally, it can result in absenteeism, immorality, and job dissatisfaction [10]. Our current experience with the pandemics of infectious diseases tells us that mental health disorders can be triggered by the difficulties in adjusting to workplace pressures caused by large numbers of patients, growing demands in care, changes in clinical practice, the rapid introduction of new technologies, and constant changes in work schedules [12,13]. In a study from New Zealand, it was pointed out that anxiety, stress, and poor psychological well-being were higher at the first testing time point in the evaluated group of nurses. At the second testing time point, it was shown that anxiety levels increased while stress levels and scores of poor psychological well-being improved [14]. The possible explanations for favorable stress and psychological well-being outcomes on the second testing point could be due to the better understanding of the SARS-CoV-2 virus over time, along with imposed measures that were implemented, which led to the reduction in COVID-19 cases. Additionally, individual factors should also be considered since it was shown that younger nurses, in contrast to their senior peers in this study, had higher levels of stress and anxiety [14]. Furthermore, the importance of work–life harmony, particularly for nurses in the COVID-19 pandemic, was pointed out in the study of Yayla and Eskici İlgin [15]. These authors found that nurses’ psychological well-being was negatively affected by the disharmony of work–life balance as well as by an increase in coronaphobia [15]. Lin and Zheng found that young age, increased weekly working time, and higher levels of anxiety were the drivers of the nurses’ stress [16]. According to the findings of the latest meta-analysis, psychological stressors due to the pandemic are negatively associated with the psychological well-being of health workers [17,18]. Since the beginning of the COVID-19 pandemic, researchers have been monitoring the mental health of health workers [19,20,21], emphasizing the need for specific strategies and programs focused on the health and well-being of all frontline health and care workers [22,23].

Given that as many as a third of patients with COVID-19 infection are hospitalized in intensive care units [24], nurses in these wards are under intense psychological pressure. Several factors that can be attributed to this include inadequate protective gear, which in the ICU might bring them close to the respiratory fluids of infected patients [25] and witnessing the highest lethality rate of critically ill COVID-19 patients, along with organizational preparedness insufficiency for the pandemic [26]. With that assumption, in 2021, we decided to investigate the perception of tension, stress, and pressure at the workplace (TSPW) among nurses in 19 health institutions with intensive care units (ICUs) in Belgrade, the capital of Serbia. 

Currently, there is insufficient knowledge regarding the psychological well-being of nurses compared to knowledge on somatic illnesses or the general population’s health. Lack of understanding of these issues often causes the risk of stigmatization [13,14,15,16,17,21]. Insufficient research in this field is putting the development of effective interventions to support the psychological health and well-being of nursing professionals in Serbia at risk. This is a critical issue, as the psychological health of nurses is directly linked to their ability to provide high-quality patient care [7,10,16]. Therefore, it is of the utmost importance to conduct further research to identify effective strategies for promoting nurses’ psychological health and well-being [23,25]. We conducted a study to test our hypothesis that most nurses working in ICUs in Serbian hospitals have experienced an increase in TSPW during the COVID-19 pandemic compared to the pre-pandemic period. Partially, this can be explained by their continuous and direct contact with COVID-19 patients, often without adequate protective gear [25]. Furthermore, these nurses are responsible for the care of critically ill COVID-19 patients and often witness fatal outcomes due to a lack of organizational preparedness for the pandemic [26]. The decision to hire an additional 2000 workers (physicians and nurses) during the pandemic [27] brought young and less experienced workers to help treat COVID-19 patients [28]. Previous crises have shown that increased stress levels, emotional burnout, and exhaustion at work are expected [29]. It is crucial to recognize if the COVID-19 pandemic has similar effects in our settings and take the necessary actions to prevent negative consequences from worsening. 

The objective of the study was to determine the extent to which ICU nurses perceive changes in their level of TSPW experienced before and during the COVID-19 pandemic. Additionally, we examined whether certain characteristics of the nurses, their perceived job satisfaction, and workplace challenges were associated with increased perceived TSPW. The study’s findings will shed light on the mental well-being of nurses working on the frontlines of the pandemic. 

## 2. Methods

### 2.1. Study Design and Participants

This multicenter study implements a cross-sectional design to investigate the associations between the ICU nurses’ self-reported data on personal characteristics, job satisfaction, and workplace challenges and the perceived increase in TSPW before and during the COVID-19 pandemic. For this secondary analysis, we took anonymous original data from the official database of the 2021 Job Satisfaction Survey of the City Institute for Public Health of Belgrade (CIPHB). 

The CIPHB routinely administered the questionnaire to all public healthcare institutions in Belgrade, involving a total of 17,103 nurses, during one working day in December 2021 (the dates varied across the institutions according to the schedule of emergency medical assistance). Based on the names of hospitals that were included in the COVID-19 system, we were able to differentiate in the electronic database hospitals with ICUs for treating patients with COVID-19 from those without ICUs and not in the COVID-19 system. In the study, we used anonymous data from all nurses from hospitals with ICUs treating patients with COVID-19 who completed the questionnaire in 2021. Given that the study was conducted during the peak of the pandemic and estimated significant excess mortality in Serbia related to COVID-19 [30], we obtained a satisfactory overall response rate to the survey of 79.4%. In this study, we performed a secondary data analysis on a total of 4210 nurses employed in all 19 ICUs in Belgrade. The gender ratio of respondents was 4.3 women to 1 man; the majority were in the age range of 34 to 54 years, and approximately one nurse was leading the team of six members. Around one-eighth of the participants have a dual practice (secondary job).

The Ethics Committee of the CIPHB approved the conduct of the study (Decision 86/1, 13 April 2022).

### 2.2. Study Instrument and Participant Variables

The study instrument was the Job Satisfaction Questionnaire, comprising 26 multiple-choice questions (https://www.batut.org.rs/download/uputstva/2021/UpinikBolnice2021.pdf) (Accessed on 2 December 2021). This instrument was created by the Institute of Public Health of Serbia “Dr Milan Jovanović Batut” to be applied annually in the National Network of all public health institutions. 

For this study, we selected input data relevant to the research questions and divided them into three groups. The first group was about nurses’ characteristics: sex (dichotomous variable, male or female), age (categorical variable, ≤34 years, 35–54 years, ≥55 years), managerial function (dichotomous variable, yes or no), and engagement in dual practice (dichotomous variable, yes or no). The second group of data displayed nurses’ satisfaction with job characteristics (categorical variables): adequacy of work equipment, adequacy of workspace, available time for work, workplace autonomy/possibility of independent decision-making, superior’s appreciation and respect, cooperation with colleagues, relation with patients, opportunity for professional development/continuous education, financial compensation for work, institutional management and work organization, adequacy of workplace hygiene, and implementation of COVID-19 infection prevention and control measures. Respondents’ satisfaction was measured on a 5-point Likert scale, where one represented “totally unsatisfied” and five represented “totally satisfied” (Cronbach’s alpha was 0.94). The third group of questions was about the work experience in COVID-19 infectious zones as well as workplace challenges during the COVID-19 pandemic: new working conditions, exhaustion due to the volume of work, exhaustion due to work in personal protective equipment, inadequate availability of personal protective equipment, inadequate access to information, uncertainty and fear of infection, and coping with patients’ feelings. Nurses were asked to mark the challenges they have faced personally (dichotomous variables); the marked question was interpreted as “yes” and the unmarked as “no”. 

In our study, the main outcome variable of interest was an increase in the value of perceived TSPW before and during the COVID-19 pandemic. Therefore, the following questions were used for nurses to self-report the level of TSPW before and during the COVID-19 pandemic:

○ “How tense, stressed, or pressured did you feel while doing your job regularly before the pandemic?”
○ “How tense, stressed, or stressed did you feel while working during the COVID-19 epidemic?”

To both questions, nurses were asked to choose one among possible answers, provided on a Likert scale from 1 to 5, where 1 was “none”—1, 2: “a little”, 3: “moderate”, 4: “a lot”, and 5: “very much” (Cronbach’s alpha was 0.83). 

The response rate to these questions was high, i.e., the missing data was 1% for TSPW before and 1.3% during the COVID-19 pandemic, and the overall range of missing data was 0–7.4%.

### 2.3. Data Processing and Statistical Analysis

For the secondary data analysis, we used descriptive statistical methods (absolute number and percentage), statistical methods for testing hypotheses, and modeling the relationship between the outcome variable and potential predictors. The validity and internal variability of the survey questions on nurses’ satisfaction with job characteristics and the main outcome variable of interest were tested with Cronbach’s alpha.

The statistically significant differences were set at *p* < 0.05 for the variables tested with Chi-square (categorical or dichotomous input variables) or Mann–Whitney (5-point Likert scale input variables). 

Wilcoxon’s rank test was used to test the difference between the perceived degrees of TSPW before and during the COVID-19 pandemic. The difference of ranks ≥1 was considered “an increase” in TSPW, and the difference ≤0 was considered “no increase”. The Wilcoxon test was statistically significant if the *p*-value was <0.05.

The work challenges nurses perceived during the COVID-19 pandemic were included in the modeling as dichotomous variables with possible “yes/no” answers, where the reference category was “no”.

A multivariate logistic regression with an odds ratio (OR) and the corresponding 95% confidence interval (CI) was used to model the relationship of the outcome variable (an increase in the degree of perceived TSWP) to those input variables previously tested as significant. While modeling, job satisfaction with workplace characteristics was observed as a discrete variable on a 5-point scale, so that each degree on a scale from 1 to 5 is compared with the following. 

All data analyses were processed in the IBM SPSS Statistics 22 (SPSS Inc., Chicago, IL, USA) software package (IBM Corp., Armonk, NY, USA, 2017) [31] or the R software environment (R Development Core Team, 2019, Vienna, Austria) [32].

## 3. Results

### 3.1. Nurses’ Perception of Tension, Stress, and Pressure at Work before and during the COVID-19 Pandemic—Main Outcome Variable of Interest

Only a small number of nurses (less than 15%) included in this study had no perception of TSPW. At the same time, the rest of the respondents perceived an increase in TSPW on a scale from the degree of “somewhat” to either “a lot” or “very much”, and this increase was statistically significant (Wilcoxon test Z = −8.327, *p* < 0.001). 

**Figure 1:**
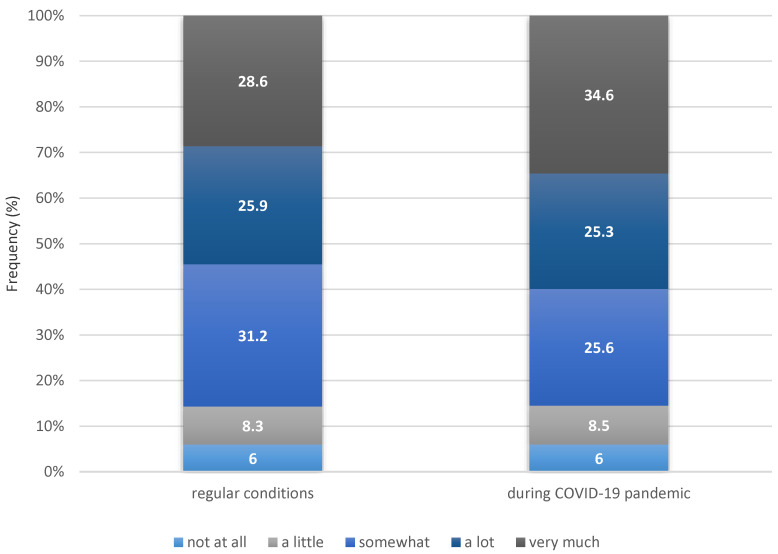
Frequency (%) of the nurses’ perception of tension, stress, and pressure at work before and during the COVID-19 pandemic.

A total of 951 nurses (22.6%) reported an increase in perceived TSPW during the COVID-19 pandemic compared to the period before the pandemic. More nurses reported “very much” when answering about their perception of TSPW during the COVID-19 pandemic (34.6%) than when they recalled their experience from before the pandemic (28.6%) (Figure 1).

### 3.2. Nurses’ Characteristics and an Increase in Perceived Tension, Stress, and Pressure at Work before and during the COVID-19 Pandemic

The majority of nurses who participated in this study were women (75.0%), belonging to the age group from 35 to 54 years (54.5%); some (13.1%) of them were managers, and some (12.3%) had dual practice, but none of those characteristics were related to an increase in TSPW (Table 1). 

### 3.3. Nurses’ Job Satisfaction in Relation to the Increase in Perceived Tension, Stress, and Pressure in the Workplace before and during the COVID-19 Pandemic

Nurses were the least satisfied (marking “bad” or “very bad” on the questionnaire) with financial compensation for their work (48.1%), the management and organization of work in their institution (27.6%), the adequacy of their workspace (25.6%), and the opportunity for professional development/continuing education (24.9%). 

However, only a few variables were associated with an increase in TSPW, including management and organization of work in the institution, available time to work, autonomy in the workplace, superiors’ appreciation and respect, cooperation with colleagues, and relation to patients’ expectations (Table 2, Supplemental File S1).

### 3.4. Challenges Related to the COVID-19 Pandemic and the Increase in Perceived Tension, Stress, and Pressure in the Workplace among Nurses 

More than two-thirds of the nurse respondents (69.9%) have performed triage, admission, and treatment tasks in the zones of ICU departments for patients infected with the SARS-CoV-2 virus. Nurses with a perceived increase in TSPW more frequently reported challenges related to the tasks in the COVID-19 zone, such as exhaustion due to working under personal protective equipment, exhaustion due to the volume of work, uncertainty and fear of infection, and coping with the patients’ feelings (Table 3).

### 3.5. Factors Associated with an Increase in Perceptions of Tension, Stress, and Pressure during Work in the Survey Population before and during the COVID-19 Pandemic

The statistically significant multivariate logistic regression model (*p* < 0.001) included 743 nurses with the study outcome of interest from 19 healthcare institutions with ICUs in Belgrade in 2021 (Table 4). 

**Figure 2:**
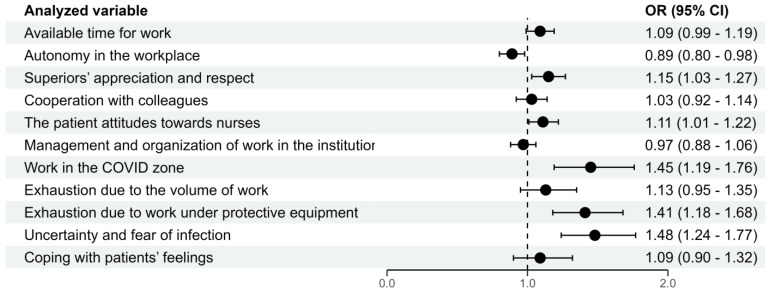
Potential predictors of increased tension, stress, and pressure during work before and during the COVID-19 pandemic in the studied nurse population (multivariate logistic regression).

The study shows that the perceived TSPW among nurses has a direct correlation with several factors. With each higher degree of supervisors’ respect and satisfaction with their relation to patients’ expectations, the perceived TSPW was likely to increase by 15% and 11%, respectively. When nurses were engaged in tasks in the COVID-19 zone, the perceived TSPW increased by 45%. The perceived TSPW also increased by 42% for those exhausted from working under personal protective equipment and by 48% for those who felt uncertainty and fear of infection. On the other hand, the perceived TSPW was likely to decline by 12% with each degree of autonomy in decision-making. These findings on potentially predictive variables can help healthcare organizations improve the well-being of their nurses and reduce the perceived TSPW (Figure 2). 

## 4. Discussion

To our knowledge, this was the first study that analyzed the TSPW perceived by nurses working in ICUs before and during the COVID-19 pandemic in Belgrade (Serbia), the capital of a middle-income country in the Western Balkans region. In that regard, its findings improve our understanding of ICU nurses’ situations, mainly the mental health needs of those on the frontline of defense against the pandemic. The study’s findings provide important insights into how to prevent nursing challenges in future outbreaks. 

A higher degree of perceived TSPW was registered in about 30% of the respondents, and it was confirmed that both the characteristics of the job and the challenges of the COVID-19 pandemic were statistically significant predictors of its occurrence. This finding was in line with the previous research on workplace stress among medical workers [33,34]. The study on Australian nurses from New South Wales during the COVID-19 pandemic noted that approximately one-fifth had higher levels of COVID-19 pandemic-related stress [35]. A lower degree of work autonomy, a higher degree of supervisors’ respect, and a higher nurse relation to patients’ expectations increased the chances for greater TPSW during the pandemic. In the study of Labrague et al., personal resilience, organizational support, and social support correlate negatively with COVID-19 anxiety among nurses [36]. Work in the COVID-19 infectious zones, exhaustion from work under personal protective equipment, and uncertainty and fear of infection were also positively associated with increased TPSW. Similarly, past research suggests that epidemics of infectious diseases increase nurses’ stress more than other health workers [8,9], and that was also confirmed during the COVID-19 pandemic [20,37].

As noted in other research [38,39,40,41], in our model, medical nurses working in the COVID-19 infectious zones were 45% more likely to have increased TPSW than those not in direct contact with COVID-19 patients. Among the challenges of working during the COVID-19 pandemic, the increase in TPSW was significantly influenced by the uncertainty, fear of infection, and exhaustion due to working under personal protective equipment. These results are comparable to the findings from previous epidemics [8,42] and research about COVID-19 [2,20,43,44], which showed that uncertainty and fear of infection negatively affect the mental health of health workers. In models that did not find increased TSPW in the first-line responders [45,46,47], adequate training and preparation were listed as factors that preceded going to COVID-19 infectious zones. Also, according to a survey, clear communication and consistent information are among the most common requirements of ICU employees [48].

According to Tabah et al., 69–87% of respondents experienced adverse effects from working while wearing protective equipment [49]. Meanwhile, Dong et al. suggest that wearing N95 masks and protective suits can interfere with interpersonal communication, resulting in negative moods or repressed emotions [37]. Likewise, our study shows that working under protective equipment increased the perceived TSPW of nurses in the ICU.

Regarding job satisfaction, in our model, a significant increase in perceived TSPW was influenced by a higher degree of respect for work by superiors, a lower degree of work autonomy, and a higher degree of patient-to-nurse relations. Indeed, other researchers have shown that high job demands and little decision-making power are associated with the development of stress-related symptoms [50]. In line with social exchange theory, employees who feel that their organization values their contributions are more likely to work harder to aid the organization, achieve the expected performance rewards, and fulfill their emotional needs [51]. On the other hand, social cognitive theory emphasizes the role of coping self-efficacy in managing stress. People who believe they can control stressful situations are less likely to be distressed. Conversely, individuals with low self-efficacy tend to overestimate threats and worry about negative outcomes [52,53,54]. In previous research [44], a higher level of autonomy was seen as a protective factor for burnout symptoms. Although each health worker was given a higher level of independence during the COVID-19 pandemic, it is assumed that the new situation needed to be more specific and structured to reduce stress and burnout symptoms. In the future study, it is necessary to examine whether nurses in managerial positions (about 14% of our sample) have actively participated in their institution’s decision-making, planning, and organization.

This study has several implications for policy and practice. Our findings indicated that interventions at the individual, institutional, and systemic levels must be prioritized to protect the mental health of nurses during a medical crisis. Individual-level interventions include establishing a solid social support system and good infrastructure that lower nurses’ stress levels and prevent burnout in crises [40,44]. Nurses in the ICU experience less stress when they have regular communication with other departments to address any uncertainties that may arise during their clinical work. Continuous education in emotional intelligence has been recognized as a valuable tool for improving teamwork and aiding patients in coping with their experiences [55]. This is particularly relevant during the pandemic, as emotional intelligence has been shown to be strongly associated with successful psychological adjustment and the avoidance of negative behaviors. This, in turn, reduces the likelihood of developing depression and anxiety [56]. Additionally, adequate personal protective equipment should be procured. Social support is also crucial, such as collaborating with social services to take care of the health workers’ families. At the institutional level, it is recommended to provide daily psychological counseling and weekly stress level check-ups for nurses working in intensive care units. It is important to maintain and improve the quality and safety of working conditions within the institution. This can be achieved by implementing regulations related to working hours, such as limiting the amount of time spent in infectious zones. At the level of health system policy, job dissatisfaction should be considered a top priority problem on the stakeholders’ agenda, as it is positively correlated with the desire to quit the job [57].

Due to the study’s cross-sectional design, we could not determine the causal relationship between the variables. Still, among the tested 24 factors, we identified potential predictors of increased TSPW perceived among nurses working at the frontline in intensive care facilities. We failed to account for certain variables that may have affected how nurses perceived TSPW, such as their experience, attitudes, motives, and personal situations, including social and economic factors and family responsibilities. However, similar to prospective or longitudinal studies [58,59], in our one-point-in-time survey, nurses self-reported on their mental health issues over two periods. However, unlike clinical measurements, some degree of underestimation or overestimation of results due to selective non-response or biases is expected in surveys. For example, an event just before the survey may have influenced the respondents’ answers. Although we cleaned the data to correct errors, duplicates, and inconsistencies in the datasets, validating the nurses’ psychometric characteristics could help better interpret the findings [60]. Since the survey was administered routinely, nurses who were displaced, worked remotely, or temporarily averted from work (due to SARS-CoV-2 infection or any other reason) might not have completed the questionnaire. Therefore, these study findings should not be generalized to other settings or to all healthcare workers. This generalization could have been attempted by extending the time of the survey to cover the total population of nurses. An alternative approach could be applying the specifically designed instrument to a representative sample of nursing professionals in the setting.

In future studies, our findings can assist in designing research instruments specific to nursing workplaces and study questions. In that regard, our study reinforces that designing a standardized questionnaire for nurses exposed to work in infectious zones is necessary, which could help bypass most of the data limitations [61,62]. It would be beneficial for future research to conduct an analysis of the TSPW of various categories of healthcare workers in hospitals, along with the hospital’s overall performance. This analysis could help identify necessary changes, including improvements in productivity, quality of care, and safety practices in the hospital work environment.

## 5. Recommendations for ICU Nurses to Better Prepare for Epidemics

The results of our study on hospital nurses’ mental health have practical implications for organizational improvement. The situation we have described might not be unique to Serbia, and our approach and findings could be relevant to many low- and middle-income countries facing similar financial constraints and consequences. Good practices and programs from more developed environments should be culturally adapted and tested before being implemented in Serbia. In this sense, our research provides a reasonable basis for evaluating such efforts when they are invested in improving the mental health of nurses working in intensive care units.

ICU nurses are at the forefront of highly stressful situations, taking on the responsibility of caring for patients and their families. Despite the emotional toll, they work tirelessly to save lives. Nurses may avoid discussing psychological issues and the medication they use to work due to perceived stigma from colleagues and management [61]. It is necessary to establish individual and organizational support to help them overcome common professional stressful psychological and somatic symptoms such as headaches, insomnia, fatigue, despair, lower back pain, and mood swings [63].

During the pandemic, nurses face increased stress due to higher risks of infection, especially with new strains of pathogens and epidemics [25]. It is crucial for nurse managers to provide proper protective equipment to ensure safe nursing practice.

Increased agitation in healthcare settings may lead to physical and psychological assaults and threats against healthcare workers [64]. Nurse managers and the organization are responsible for establishing safety policies and procedures to ensure the safety of all parties involved [61]. Recent studies also show the benefits of educational programs on self-protection in the workplace [65].

Nurses may also be stressed when dealing with cases of adverse patient outcomes, patients’ attitudes toward them, and potential victimization [61,66]. Nurses need safe zones to communicate concerns and fear of making mistakes [61].

The nurses’ psychological well-being was negatively affected by the disharmony of work–life balance as well as by an increase in coronaphobia [15]. Nurses reported fatigue, particularly when they had to combine shifts, work long hours at night or during holidays, and were compulsorily relocated to other teams to fill the gaps due to the lack of personnel [63]. This was important since the global shortage of nurses was reported before, throughout, and in the aftermath of the COVID-19 pandemic [67].

Regarding personal characteristics, the working experience of nurses is an important factor since more experienced nurses report greater self-compassion associated with less pandemic-related stress, greater posttraumatic growth, and better psychological adjustment outcomes [35]. Therefore, when delegating tasks in unfamiliar circumstances, it is essential for those in charge to carefully analyze the restructuring of job positions, the distribution of workloads, the establishment of safety procedures, and a collaborative work environment. Additionally, it is important to assess the health capabilities, competency, and experience of nurses when dealing with intricate patient cases, as well as their knowledge and proficiency in operating new equipment.

## 6. Conclusions

Nurses in the ICUs in Belgrade, Serbia, perceived significantly increased levels of tension, stress, and pressure at work during the COVID-19 pandemic compared to their recollection of the period before the pandemic. The increase was associated with work conditions in the infectious zones, exhaustion from work under personal protective equipment, uncertainty and fear of infection, satisfaction with superiors’ respect and patients’ attitudes, and a lower degree of work autonomy. The findings of the study indicate that it is essential to find a solution to tackle the issue of ensuring the safety of nurses while they wear protective equipment for extended periods. Nurses, who are working on the frontlines of the pandemic, need better health protection, improved working conditions, and respect for their critical role. They face significant physical and mental strain, and it is crucial to address their concerns to enable them to perform their duties efficiently. A transparent chain of command and responsibility, respect for their role, and strategies to promote mental health would help reduce nurses’ stress and increase job satisfaction. Creating a standardized questionnaire for nurses who work in infectious zones is necessary to protect their mental health during emergencies.

## Figures and Tables

**Table 1:** Nurses’ characteristics in relation to the increase in perceived tension, stress, and pressure at work before and during the COVID-19 pandemic.

Individual Characteristics	Total *n* = 4210 (100%)	Increase in TSPW *n* = 951 (22.6%)	No Increase in TSPW *n* = 3203 (77.1%)	*p*
Sex, *n* (%)				0.219 ^a^
Male	743 (17.6%)	184 (19.3%)	559 (17.5%)	
Female	3156 (75.0%)	715 (75.5%)	2441 (76.2%)	
missing	311 (7.4%)	52 (5.5%)	203 (6.3%)	
Age, *n* (%)				0.648 ^b^
≤34	1174 (27.9%)	252 (26.5%)	922 (28.8%)	
35–54	2295 (54.5%)	553 (58.1%)	1742 (54.4%)	
≥55	646 (15.3%)	137 (14.4%)	509 (15.9%)	
missing	95 (2.3%)	9 (0.9%)	30 (0.9%)	
Managerial function, *n* (%)				0.876 ^a^
Yes	552 (13.1%)	125 (13.1%)	427 (13.3%)	
No	3408 (81.0%)	782 (82.2%)	2626 (82.0%)	
missing	250 (5.9%)	44 (4.6%)	150 (4.7%)	
Dual practice, *n* (%)				0.821 ^a^
Yes	511 (12.1%)	119 (12.5%)	392 (12.2%)	
No	3643 (86.5%)	832 (87.5%)	2811 (87.8%)	
missing	56 (1.3%)	0 (0%)	0 (0%)	

Legend: TSPW stands for tension, stress, and pressure in the workplace; ^a^ Chi-square test; ^b^ Mann–Whitney test.

**Table 2:** Nurses’ satisfaction with the significant characteristics of the workplace in relation to the increase in perceived tension, stress, and pressure at the workplace before and during the COVID-19 pandemic.

Satisfaction with the Characteristics of the Workplace (1—Totally Unsatisfied, 5—Totally Satisfied)	Total *n* = 4210 (100%)	Increase in TSPW *n* = 951 (22.6%)	No Increase in TSPW *n* = 3203 (77.1%)	Mann–Whitney Test, *p*
Available time for work, *n* (%)				
1	318 (7.8%)	43 (4.6%)	275 (8.7%)	<0.001
2	401 (9.8%)	79 (8.4%)	322 (10.2%)	
3	981 (24.0%)	218 (23.2%)	763 (24.3%)	
4	1140 (27.9%)	286 (30.4%)	854 (27.1%)	
5	1247 (30.5%)	315 (33.5%)	932 (29.6%)	
Autonomy in the workplace, *n* (%)				
1	445 (10.9%)	73 (7.9%)	372 (11.8%)	<0.001
2	479 (11.8)	89 (9.7)	39 0 (12.4%)	
3	1000 (24.6%)	219 (23.8%)	781 (24.8%)	
4	1141 (28.0%)	313 (34.0%)	828 (26.3%)	
5	1007 (24.7%)	227 (24.6%)	780 (24.8%)	
Superiors’ appreciation and respect, *n* (%)				
1	503 (12.2%)	59 (6.3)	444 (14.0%)	<0.001
2	487 (11.9%)	103 11.0%)	384 (12.1%)	
3	829 (20.2%)	197 (21.0%)	632 19.9%)	
4	1039 (25.3%)	255 (27.2%)	784 (24.7%)	
5	1251 (30.4%)	323 (34.5%)	928 (29.3%)	
Cooperation with colleagues, *n* (%)				
1	130 (3.2%)	21 (2.2%)	109 (3.4%)	<0.001
2	199 (4.8)	26 (2.8%)	173 (5.5%)	
3	635 (15.5%)	128 (13.6)	507 (16.0%	
4	1354 (33.0%)	329 (35.0%)	1025 (32.4%)	
5	1787 (43.5%)	436 (46.4%)	1351 (42.7%)	
The patients’ attitudes toward you, *n* (%)				
1	215 (5.4%)	26 (2.8%)	189 (6.2%)	0.003
2	244 (6.2%)	40 (4.4%)	204 (6.7%)	
3	733 (18.5%)	175 (19.1%)	558 (18.3%)	
4	1227 (30.9%)	297 (32.5%)	930 (30.5%)	
5	1546 39.0%)	377 (41.2%)	1169 (38.3%)	
Management and organization of work in the institution, *n* (%)				
1	533 (13.2%)	71 (7.7%)	462 (14.8%)	0.003
2	586 (14.5%)	139 (15.1%)	447 (14.3%)	
3	989 (24.4%)	235 (25.5%)	754 (24.1%)	
4	984 (24.3%)	263 (28.6%)	721 (23.0%)	
5	959 (23.7%)	213 (23.1%)	746 (23.8%)	

Legend: TSPW stands for tension, stress, and pressure in the workplace. The missing data were not taken into consideration in the variables (all percentages are valid).

**Table 3:** Challenges related to the COVID-19 pandemic and the increase in perceived tension, stress, and pressure in the workplace among nurses.

Challenges Related to the COVID-19 Pandemic	In Total *n* = 4210 (100%)	Increase in TSPW *n* = 951 (22.6%)	No Increase in TSPW *n* = 3203 (77.1%)	Chi-Square *p*
Work in COVID-19 zone				<0.001
Yes	2585 (70.0%)	643 (76.4%)	1942 (68.2%)	
No	1106 (30.0%)	199 (23.6%)	907 (31.8%)	
Work in completely new conditions				0.234
Yes	1778 (42.8%)	423 (44.5%)	1355 (42.3%)	
No	2376 (57.2%)	528 (55.5%)	1848 (57.7%)	
Exhaustion due to work volume				0.035
Yes	1998 (48.1%)	486 (51.1%)	1512 (47.2%)	
No	2156 (51.9%)	465 (48.9%)	1691 (52.8%)	
Exhaustion due to work under protective equipment				<0.001
Yes	2075 (50.0%)	563 (59.2%)	1512 (47.2%)	
No	2079 (50%)	388 (40.8%)	1691 (52.8%)	
Inadequate availability of protective equipment				0.325
Yes	546 (13.1%)	134 (14.1%)	412 (12.9%)	
No	3608 (86.9%)	817 (85.9%)	2791 (87.1%)	
Inadequate access to information				0.521
Yes	670 (16.1%)	147 (15.5%)	523 (16.3%)	
No	3484 (83.9%)	804 (84.5%)	2680 (83.7%)	
Uncertainty and fear of infection				<0.001
Yes	1285 (30.9%)	361 (38.0%)	924 (28.8%)	
No	2869 (69.1%)	590 (62.0%)	2279 (71.2%)	
Coping with patients’ feelings				0.009
Yes	995 (24.0%)	258 (27.1%)	737 (23.0%)	
No	3159 (76.0%)	693 (72.9%)	2466 (77.0%)	

Note: The missing data were not taken into consideration in the variables (all percentages are valid). TSPW stands for tension, stress, and pressure in the workplace.

**Table 4:** Multivariate logistic regression of an increase in tension, stress, and pressure perceived by nurses from 19 healthcare institutions with ICUs in Belgrade in 2021.

Variables	Multivariate Logistic Regression, *n* = 743 (Reference = 1, No Increase in TSPW)				
	B	*p*	Odds Ratio (OR)	95% Confidence Interval	
				Lower Limit	Upper Limit
Available time for work	0.085	0.069	1.089	0.993	1.194
Autonomy in the workplace	0.118	0.021	0.889	0.804	0.982
Superiors’ appreciation and respect	0.138	0.010	1.147	1.034	1.274
Cooperation with colleagues	0.027	0.617	1.027	0.924	1.142
The patients’ attitudes	0.105	0.023	1.111	1.015	1.216
Management and organization of work in the institution	0.034	0.474	0.967	0.881	1.061
Work in the COVID zone					
Yes	0.369	<0.001	1.446	1.188	1.760
No			1		
Exhaustion due to the volume of work					
Yes	0.124	0.161	1.132	0.952	1.347
No			1		
Exhaustion due to work under protective equipment					
Yes	0.345	<0.001	1.413	1.184	1.685
No			1		
Uncertainty and fear of infection					
Yes	0.392	<0.001	1.481	1.241	1.767
No			1		
Coping with patients’ feelings					
Yes	0.091	0.352	1.095	0.905	1.325
No			1		

Legend: TSPW stands for tension, stress, and pressure in the workplace. There was no multicollinearity between the variables (VIF range 1.1–2.8).

## Data Availability

The data presented in this study are available on request from the corresponding author.

## Supplementary Materials

The following supporting information can be downloaded at https://www.mdpi.com/article/10.3390/healthcare12060663/s1, Supplemental File S1. Nurses’ satisfaction with the characteristics of the workplace in relation to the increase in perceived tension, stress and pressure at the workplace before and during the COVID-19 pandemic.
