# Supplementary material for: Nurses’ Perception of Tension, Stress, and Pressure before and during the COVID-19 Pandemic: A Multicenter Serbian Study
Source: Healthcare (Basel). 2024 Mar 15;12(6):663. doi: 10.3390/healthcare12060663 (PMC10969813; doi:10.3390/healthcare12060663)
Supplement: Supplementary file 1 [file healthcare-12-00663-s001.zip › healthcare-2884521-supplementary.pdf]

Supplemental file S1. Nurses' satisfaction with the characteristics of the workplace in relation to the increase in perceived tension, stress and pressure at the workplace before and during the COVID-19 pandemic.

Satisfaction with the characteristics of the workplace (1-very bad, 5-excellent)	Total n=4210 (100%)	Increase in TSPW n=951 (22.6%)	No increase in TSPW n=3203 (77.1%)	p[b]
1. Adequacy of work equipment, n (%)				
1	300 (7.3 %)	51 (5.4%)	249 (7.8 %)	
2	455 (11.0 %)	95 (10.0 %)	360 (11.3 %)	
3	1128 (27.4 %)	275 (29.1 %)	853 (26.9 %)	
4	1161 (28.2 %)	298 (31.5 %)	863 (27.2 %)	0.542
5	1076 (26.1 %)	227 (24.0 %)	849 (26.7 %)	
2. Adequacy of workspace, n (%)				
1	463 (11.2%)	81 (8.6%)	382 (12.0%)	
2	590 (14.3%)	130 (13.8%)	460 (14.4%)	
3	1033 (25.0%)	250 (26.6%)	783 (24.6%)	0.221
4	1066 (25.8%)	271 (28.8%)	795 (25.0%)	
5	975 (23.6%)	209 (22.2%)	766 (24.0%)	
3. Available time for work, n (%)				
1	318 (7.8%)	43 (4.6%)	275 (8.7%)	
2	401 (9.8%)	79 (8.4%)	322 (10.2%)	
3	981 (24.0%)	218 (23.2%)	763 (24.3%)	<0.001
4	1140 (27.9%)	286 (30.4%)	854 (27.1%)	
5	1247 (30.5%)	315 (33.5%)	932 (29.6%)	
4. Autonomy in the workplace, n (%)				
1	445 (10.9%)	73 (7.9%)	372 (11.8%)	
2	479 (11.8)	89 (9.7)	39 0 (12.4%)	<0.001
3	1000 (24.6%)	219 (23.8%)	781 (24.8%)	
4	1141 (28.0%)	313 (34.0%)	828 (26.3%)	
5	1007 (24.7%)	227 (24.6%)	780 (24.8%)	
5. Superiors' appreciation and respect,				
n (%)	503 (12.2%)	59 (6.3)	444 (14.0%)	
1	487 (11.9%)	103 11.0%)	384 (12.1%)	
2	829 (20.2%)	197 (21.0%)	632 19.9%)	< 0.001
3	1039 (25.3%)	255 (27.2%)	784 (24.7%)	
4	1251 (30.4%)	323 (34.5%)	928 (29.3%)	
5				
6. Cooperation with colleagues, n (%)				
1	130 (3.2%)	21 (2.2%)	109 (3.4%)	
2	199 (4.8)	26 (2.8%)	173 (5.5%)	
3	635 (15.5%)	128 (13.6)	507 (16.0%	<0.001
4	1354 (33.0%)	329 (35.0%)	1025 (32.4%)	
5	1787 (43.5%)	436 (46.4%)	1351 (42.7%)	
7. The patients' attitudes towards you,				
n (%)	215 (5.4%)	26 (2.8%)	189 (6.2%)	
1	244 (6.2%)	40 (4.4%)	204 (6.7%)	
2	733 (18.5%)	175 (19.1%)	558 (18.3%)	0.003

3	1227 (30.9%)	297 (32.5%)	930 (30.5%)	
4	1546 39.0%)	377 (41.2%)	1169 (38.3%)	
5				
8. Opportunity for professional development, n (%)				
1	498 (12.2%)	92 (9.8%)	406 (12.9%)	
2	518 (12.7%)	115 (12.2%)	403 (12.8%)	
3	934 (22.8%)	217 (23.1%)	717 (22.7%)	0.110
4	985 (24.1%)	253 (26.9%)	732 (23.2%)	
5	1156 (28.3%)	262 (27.9%)	894 (28.4%)	
9. Financial compensation for work, n				
(%)	1127 (27.3%)	218 (23.1%)	909 (28.6%)	
1	856 (20.8%)	229 (24.3%)	627 (19.7%)	
2	925 (22.4%)	233 (24.7%)	692 (21.8%)	0.574
3	641 (15.5%)	158 (16.8%)	483 (15.2%)	
4	574 (3.9%)	105 (11.1%)	469 (14.7%)	
5				
10. Management and organization of work in the institution, n (%)				
1	533 (13.2%)	71 (7.7%)	462 (14.8%)	
2	586 (14.5%)	139 (15.1%)	447 (14.3%)	
3	989 (24.4%)	235 (25.5%)	754 (24.1%)	0.003
4	984 (24.3%)	263 (28.6%)	721 (23.0%)	
5	959 (23.7%)	213 (23.1%)	746 (23.8%)	
11. Prevention measures for nosocomial infection, n (%)				
1	381 (9.3%)	64 (6.8%)	317 (10.0%)	
2	528 (12.9%)	113 (12.0%)	415 (13.1%)	0.094
3	933 (22.7%)	217 (23.0%)	716 (22.7%)	
4	1096 (26.7%)	293 (31.0%)	803 (25.4%)	
5	1165 (28.4%)	257 (27.2%)	908 (28.7%)	
12. Prevention measures for COVID-19 infection, n (%)				
1	340 (8.3%)	54 (5.7%)	286 (9.1%)	
2	420 (10.2%)	97 (10.3%)	323 (10.2%)	0.114
3	891 (21.7%)	205 (21.7%)	686 (21.8%)	
4	1095 (26.7%)	277 (29.3%)	818 (25.9%)	
5	1352 (33.0%)	311 (32.9%)	1041 (33.0%)	

Legend: TSPW stands for tension, stress, and pressure in the workplace. The missing data were not taken into consideration in the variables (all percentages are valid). [b] Mann Whitney test
